# A parametric study of the hydrodynamic roughness produced by a wall coating layer of heavy oil

**DOI:** 10.1007/s12182-016-0144-z

**Published:** 2017-01-16

**Authors:** S. Rushd, R. S. Sanders

**Affiliations:** Department of Chemical and Materials Engineering, University of Alberta, Edmonton, AB Canada

**Keywords:** Pipeline transportation, Heavy oil, Wall fouling, Lubricated pipe flow, CFD simulation

## Abstract

In water-lubricated pipeline transportation of heavy oil and bitumen, a thin oil film typically coats the pipe wall. A detailed study of the hydrodynamic effects of this fouling layer is critical to the design and operation of oil–water pipelines, as it can increase the pipeline pressure loss (and pumping power requirements) by 15 times or more. In this study, a parametric investigation of the hydrodynamic effects caused by the wall coating of viscous oil was conducted. A custom-built rectangular flow cell was used. A validated CFD-based procedure was used to determine the hydrodynamic roughness from the measured pressure losses. A similar procedure was followed for a set of pipe loop tests. The effects of the thickness of the oil coating layer, the oil viscosity, and water flow rate on the hydrodynamic roughness were evaluated. Oil viscosities from 3 to 21300 Pa s were tested. The results show that the equivalent hydrodynamic roughness produced by a wall coating layer of viscous oil is dependent on the coating thickness but essentially independent of oil viscosity. A new correlation was developed using these data to predict the hydrodynamic roughness for flow conditions in which a viscous oil coating is produced on the pipe wall.

## Introduction

The reserves of non-conventional oils, such as heavy oil and bitumen, form a substantial part of the known global petroleum resources (IEA 2014; CAPP 2014). These oil reserves are asphaltic, dense, and highly viscous, with bitumen being more dense and viscous than heavy oil (Saniere et al. 2004). Densities of these oils are nearly the same as that of water, whereas their viscosities are higher than that of water by orders of magnitude (McKibben et al. 2000). Therefore, the production of these non-conventional oils requires extraordinary techniques that are not needed to recover traditional petroleum deposits. Various mining and in situ production technologies are being used to extract non-conventional oils in Canada. After extraction, the oil is transported from a production site to an upgrading facility. Since pipeline transportation is a cost-effective technology, the non-conventional oil industry is keen to use this technology for transporting both bitumen and heavy oil (Nunez et al. 1998; Saniere et al. 2004; Hart 2014).

**Fig. 1:**
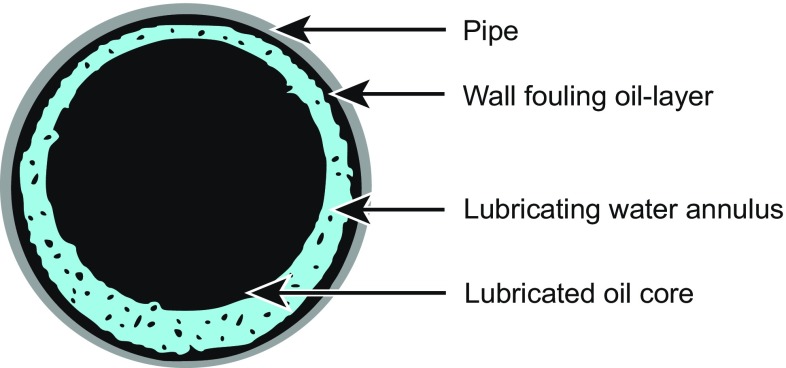
Illustration of the flow regime in a LPF pipeline

Water-lubricated pipeline transportation of non-conventional oils, known as lubricated pipe flow (LPF), is one option for transporting these viscous fluids. It is more economical when compared with other technologies, such as heating, solvent dilution, emulsification, and partial upgrading (Nunez et al. 1998; Saniere et al. 2004). In LPF, a thin water annulus prevents continuous contact between the pipe wall and the viscous oil core, resulting in much lower energy requirements than would be needed to transport the viscous oil alone in the pipeline (Arney et al. 1993; Joseph et al. 1999; McKibben et al. 2000; Rodriguez et al. 2009; de Andrade et al. 2012). The water could be naturally present in the oil or could be injected for the purpose of producing LPF. A concern for the application of LPF is the formation of an oil film on the wall (Nunez et al. 1998; Saniere et al. 2004). This oil layer is usually identified as “wall fouling.” The probable flow regime in LPF is schematically presented in Fig. 1. In this figure, a fouling oil layer is shown to surround a thin water annulus that lubricates the oil-rich core. The mechanism of wall fouling has not previously been studied in any detail. Previous experimental works suggest the fouling layer is a natural consequence of the lubrication process (Joseph et al. 1999; Vuong et al. 2009). Frictional pressure losses in a fouled pipe are much higher (say, 8–15 times) than those measured in an unfouled pipe (Arney et al. 1996), but still orders of magnitude lower than those would be expected for transporting only heavy oil or bitumen. It has been found in repeated tests that the formation of this wall coating is practically unavoidable in the industrial application of LPF technology (McKibben et al. 2000, 2016). Different degrees of wall fouling occur depending on the conditions of LPF, e.g., water cut, oil viscosity, and superficial velocity (Joseph et al. 1999; Schaan et al. 2002; Rodriguez et al. 2009; Vuong et al. 2009).

The wall fouling layer in a water-lubricated pipeline can be considered as a stationary coating film of viscous oil adhered on the pipe wall. This is because the relative velocity of this layer is negligible compared to the average mixture velocity (Joseph et al. 1999; McKibben et al. 2000, 2016; Shook et al. 2002; Schaan et al. 2002; Vuong et al. 2009). This wall coating layer can produce a large equivalent hydrodynamic roughness value: The typical equivalent roughness of a commercial steel pipe is about 0.045 mm, while the hydrodynamic roughness (inferred from pressure loss measurements) of a pipeline with a viscous oil layer on the pipe wall can be greater than 1 mm (Brauer 1963; Shook et al. 2002). The roughness is produced primarily through contact between the viscous oil coating and the turbulent water layer that flows over the film while lubricating the oil core. The result is a rippled/rough wall that is associated with very large hydrodynamic roughness values (Brauer 1963; Picologlou et al. 1980; Shook et al. 2002). While the presence of the coating reduces somewhat the cross-sectional area available for flow, which also causes an increase in pressure loss for a given throughput, the increased hydrodynamic roughness plays a much more important role in this increase.

The conventional method for describing the hydrodynamic roughness produced by a rough solid wall is the Nikuradse sand grain equivalent (Perry and Green 1997; Whyte 1999). This definition of equivalent roughness is extensively used for commercial metal pipes or channels and has also been used to describe the hydrodynamic roughness caused by a biofilm on a solid wall (Picologlou et al. 1980). Much like an oil fouling layer, the biofilm is conformable and can substantially increase the hydrodynamic roughness, in turn increasing power requirements for pumping water through bio-fouled pipes and channels (Barton et al. 2008; Andrewartha et al. 2008).

Previous studies of equivalent hydrodynamic roughness involved either a rectangular flow cell or a pipe for experiments (Barton et al. 2008; Andrewartha et al. 2008). In rectangular flow cells, one wall is typically “roughened” (e.g., through the formation of a biofilm) while the other three walls are kept hydrodynamically smooth. The time-averaged velocity profile perpendicular to the rough wall is then measured to determine the hydrodynamic roughness on the basis of correlations, such as the law of the wall (Andrewartha et al. 2008). The reliability of the measurement was subject to the type of instrumentation selected for the measurements and also the size of the flow cell. Typically, a large channel was used to ensure that the measured velocity profile would not be affected by the presence of the walls.

Pressure loss measurements have been typically used to determine the hydrodynamic roughness for the pipeline tests using some basic equations of fluid dynamics, such as the Darcy–Weisbach equation or the Colebrook correlation (e.g., Barton et al. 2008). A basic analytical approach such is appropriate when the hydrodynamic roughness can be represented by a single, constant value. In other words, this approach is not applicable for the flow cells with asymmetric wall roughness.

In the present study, a customized rectangular flow cell was used to perform a parametric investigation of the equivalent hydrodynamic roughness produced by a wall coating of viscous oil. A relatively small test cell was chosen because the goal was to test a number of different oils under a wide range of flow conditions. As a result, it was very difficult to make accurate measurements of the velocity profile of the flow above the coated surface and instead the pressure loss (under fully developed flow conditions) was measured for the different flow conditions tested. The asymmetry of wall roughness in the flow cell (one rough wall and three smooth walls), however, meant that a simple analytical approach to relate pressure loss to hydrodynamic roughness could not be used. Therefore, the flow conditions in the experimental cell were modeled using a commercially available CFD package (ANSYS CFX 13.0) and simulations were conducted to determine the hydrodynamic roughness that would give a predicted pressure loss equal to that measured during an experiment. The validated CFD-based procedure has been described in detail elsewhere (Rushd et al. 2016). Based on the results presented here, a new correlation is proposed for the equivalent hydrodynamic roughness produced by a viscous layer of wall coating in terms of the coating thickness. This correlation can be used to estimate the hydrodynamic roughness from a measured or a known value of the physical wall coating thickness.

## Experimental setup and procedure

**Fig. 2:**
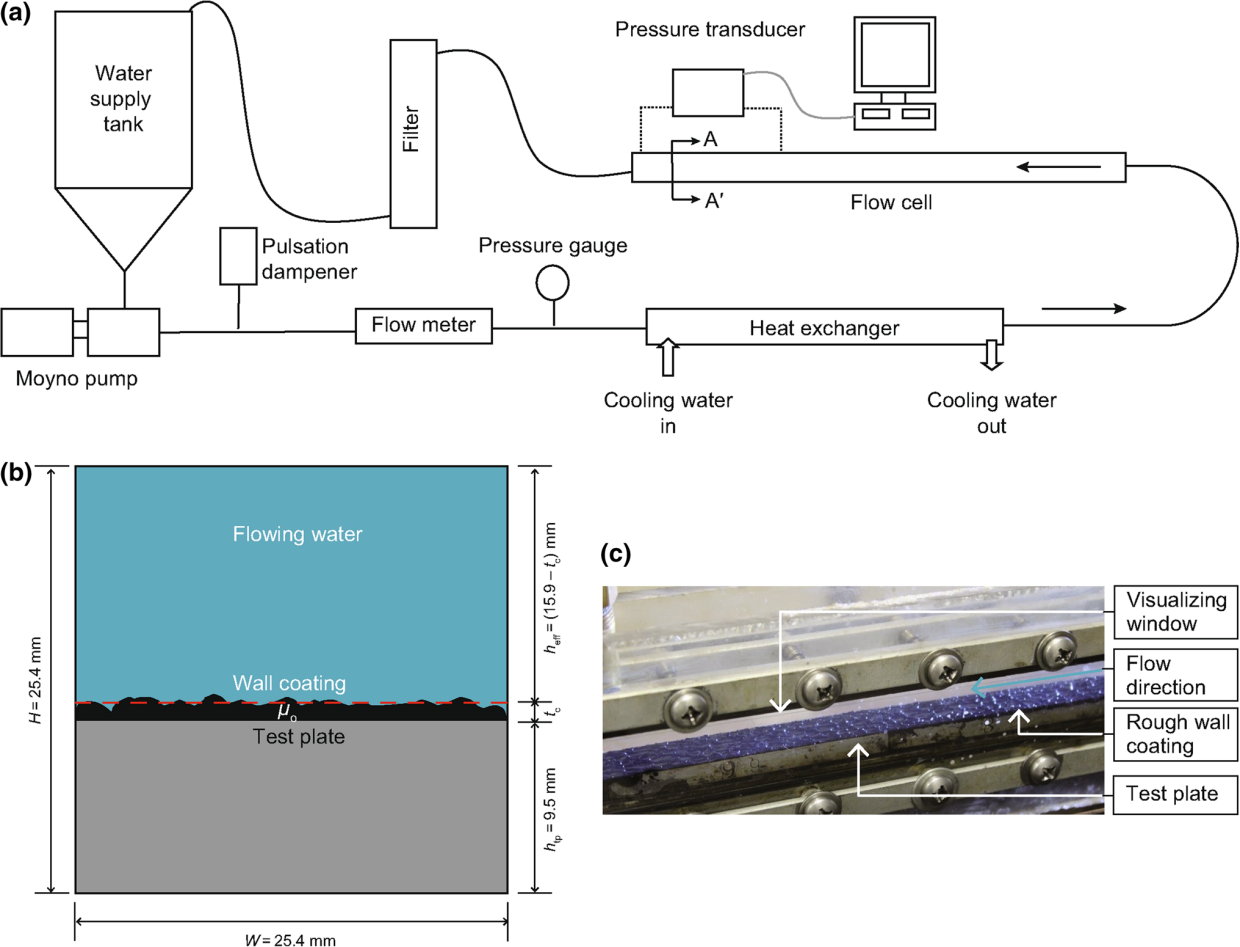
Illustration of the experimental facility. **a** Schematic presentation of the flow loop. **b** Cross-sectional view (section A–A’) of the flow cell. **c** Photograph showing a test with a rough wall coating of viscous oil (*μ*_o_ = 21300 Pa s)

A 2.5-m-long rectangular flow cell was designed and fabricated for the present study. The flow cell consisted of a channel whose lower surface was comprised of segmented steel plates. These plates were coated with a measured, constant thickness (*t*_c_) of oil prior to the start of each flow experiment. The effective cross section of the flow channel without a wall coating was 15.9 mm × 25.4 mm. Its entrance length was 1.5 m, which was more than 60*D*_h_; where *D*_h_ = 19.6 mm is the hydraulic diameter defined as 4*A*/*P*, where *A* is the cross-sectional area and *P* is the wetted perimeter of the cross-sectional area. The flow cell had two Plexiglas windows so that it was possible to observe the shape of oil–water interface. This custom-built cell was placed in a 25.4-mm pipe loop as shown in Fig. 2a. A cross-sectional view of the flow cell is presented as Fig. 2b. A photograph of the cell under actual flow conditions, after the rough/rippled topology was developed on the wall coating layer, is given in Fig. 2c.

**Fig. 3:**
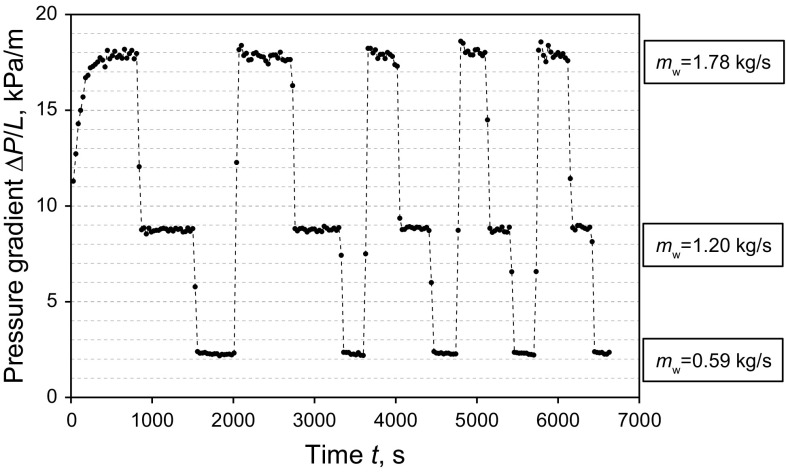
Illustration of pressure gradients (Δ*P/L*) measured over time (*t*) for different water flow rates (*m*_w_) (*t*_c_ = 0.2 mm; *μ*_o_ = 21300 Pa s)

The steady-state pressure loss across the flow cell was measured with a differential pressure transducer (Validyne P61). The experiments were conducted for a range of water flow rates, coating thickness, and oil viscosities. Typical pressure gradient measurements (30 s averages) are presented for a specific flow condition in Fig. 3. These results demonstrate that the change in pressure loss for a given water flow rate is negligible when the thickness of the wall coating layer is constant.

For experiments, each of the segmented plates comprising the bottom wall of the flow visualizing section was coated separately with a specific thickness of the viscous oil and placed in the flow cell to form a coating layer of uniform thickness. The average thickness of the coating layer (*t*_c_) was determined by weighing the test plates without and with coating oil. It should be noted that the coated plates were also weighed before and after each experiment. The difference between the measured weights was negligible, i.e., *t*_c_ was unaffected by the flow rate and thus was taken as a controlled parameter.

Please refer to Rushd (2016) for more detailed descriptions of the experimental apparatus, procedures, and results.

## Experimental parameters

The rectangular flow cell was used to study the hydrodynamic effect of different viscous wall coatings. The measured variable was the pressure loss (Δ*P*). The controlled parameters are listed in Table 1. The most important of these parameters are the average thickness (*t*_c_) and the viscosity (*µ*_o_) of the coating oil. Recall that only the bottom wall of the rectangular flow cell was coated with oil. The experimental value of *t*_c_ for an oil was selected depending on oil viscosity (*µ*_o_) and flow rate of water (*m*_w_). The thickness (*t*_c_) that could be maintained under the highest flow rate for the lower viscosity oils (*µ*_o_ ~ 65 and 320 Pa s) in the flow cell was 0.2 mm. Similarly, the maximum *t*_c_ for the higher-viscosity oils (*µ*_o_ ~ 2620 and 21300 Pa s) was 1.0 mm. Coating thickness values tested for these oils were 0.2, 0.5, and 1.0 mm. The overall uncertainty associated with the measurement of *t*_c_ in the flow cell was 10%. Thus, the coating thickness (*t*_c_) for the first phase of experiments was selected so that it would not change significantly with water flow rate. The purpose of these tests was to evaluate the effects of the flow rate and the viscosity on the hydrodynamic roughness while keeping the coating thickness constant.

**Table 1:** Controlled parameters for the rectangular flow cell experiments

Controlled parameter	Value(s)
Thickness of wall coating *t*_c_, mm	0.2, 0.5, and 1.0
Viscosity of coating oil *µ*_o_, Pa s	65, 320, 2620, and 21300
Mass flow rate of water *m*_w_, kg/s	0.59, 0.91, 1.20, 1.52, and 1.78
Water Reynolds numbers *Re*_w_	29000 < *Re*_w_ < 87000
Flow temperature *T*, °C	20

## CFD simulations

As mentioned previously, the CFD simulations were used to determine the unknown equivalent sand grain roughness (*k*_s_) of the oil-covered bottom wall of the flow cell. This was done by modeling the water flow through the cell over the viscous coating. The CFD software package, ANSYS CFX 13.0, was used for simulation. The software solves the governing differential equations that include Reynolds-averaged Navier–Stokes (RANS) continuity and momentum equations. The Reynolds stress term in RANS was modeled using an omega-based Reynolds stress model, *ω*-RSM. Full details of the governing equations are given in Appendix 1.

The geometry of the 3D computational domain used for the simulation was identical to the rectangular flow cell; however, two different flow cell lengths (*l* = 1.0 m; *l* = 2.0 m) were used for computations even though the actual flow cell was 1.0 m in length. This was done to ensure the length independence of the simulations. The width (*w*) was equal to that of the flow cell (25.4 mm). The height (*h* = 15.9−*t*_c_ mm) was varied depending on the average thickness (*t*_c_) of oil coating on the bottom wall. The values of *t*_c_ tested during the present study are shown in Table 1.

**Fig. 4:**
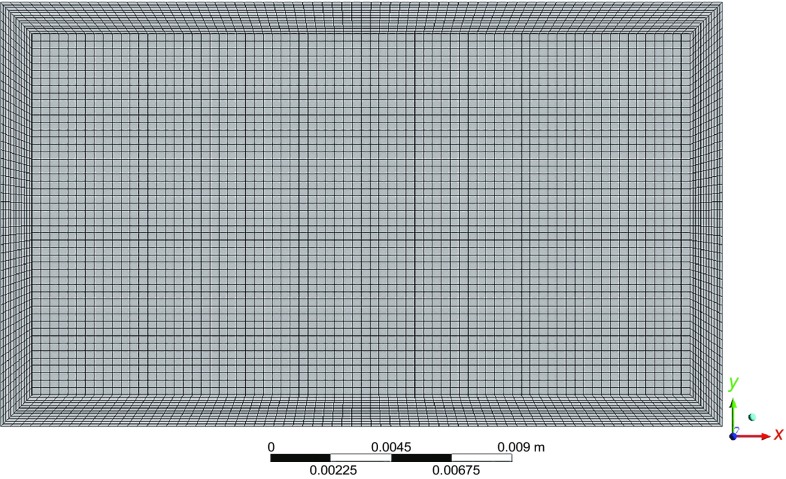
Two-dimensional illustration of the fine mesh used for simulations

The flow geometry was created and meshed with ANSYS ICEM CFD. The software was used to discretize the flow domain into structured grids, one for the bulk of the flow and one for the near-wall region. Coarse, intermediate, and fine mesh grids were tested. The mesh resolution was based on the number of nodes, *n*, in each mesh. In the present study, the mesh resolution is classified as follows: coarse (*n* < 50000), intermediate (50000 < *n* < 500000), and fine (*n* > 500000). The total number of nodes found to be sufficient for grid independence was *n* = 670200. An example of the fine mesh used here is shown in Fig. 4. The number of nodes in the near-wall region was selected so that *y* ^+^ > 11.06. At *y* ^+^ = 11.06, ANSYS CFX 13.0 uses the logarithmic law of the wall (i.e., the wall function). For these simulations, typically, *y* ^+^ = 25. All computations were performed to obtain steady-state solutions. Double precision was used in the computations, and solutions were considered converged when the normalized sum of the absolute dimensionless residuals of the discretized equations was less than 10^−6^. The typical computational time required for the convergence of a simulation was 45 min.

**Fig. 5:**
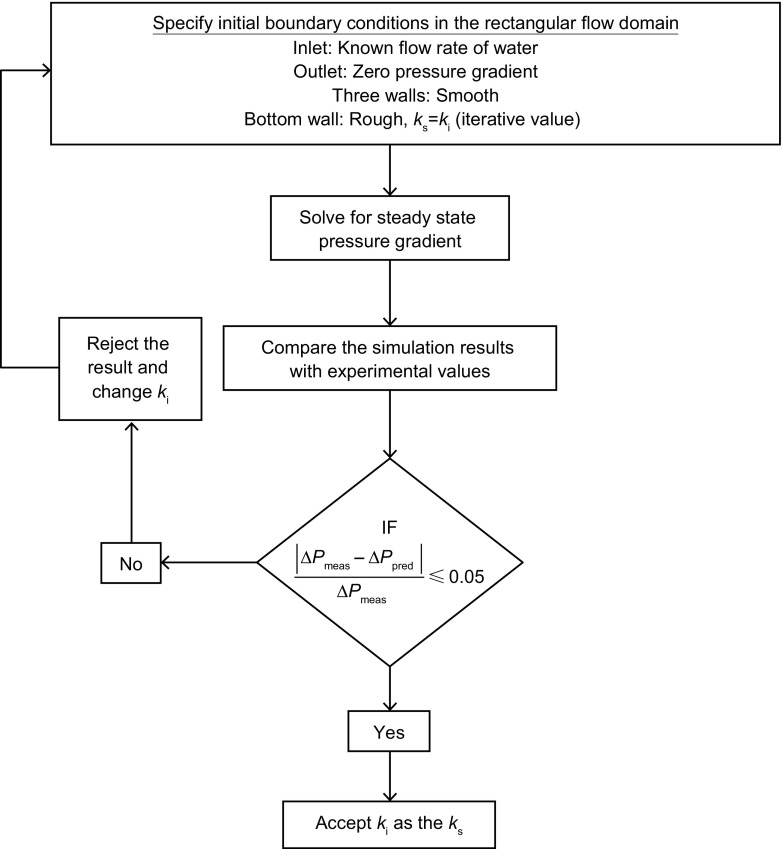
Flowchart describing the steps involved in the simulation procedure for computing the equivalent sand grain roughness (*k*_s_)

At the inlet of the flow domain, the experimental mass flow rate of water and a turbulent intensity of 5% were prescribed as the boundary condition. A zero pressure condition was specified at the outlet. The no-slip condition was used at the boundaries representing walls. The two side walls and the upper wall in the rectangular domain were taken as hydrodynamically smooth (*k*_s_ = 0) based on the results of simulations conducted for clean walls (Rushd et al. 2016). Flow conditions where the bottom wall was coated with oil required one to specify the *k*_s_ value for this wall. However, the values of *k*_*s*_ were unknown for the oil-coated bottom wall of the flow cell for any given flow condition. A trial and error procedure was adopted to determine the appropriate *k*_s_ value. Starting from a low value, *k*_s_ was increased in steps and the simulation was repeated until a reasonable agreement between the measured and predicted pressure loss (maximum 5% difference) was observed. The final value of *k*_s_ at which this condition was met was considered to be the equivalent hydrodynamic roughness of the corresponding rough wall. The trial and error approach is summarized in Fig. 5. This CFD-based trial and error approach of estimating *k*_s_ was validated using data on biofilms taken from the literature and from flow cell tests using materials of known roughness (Rushd et al. 2016).

## Results and discussion

As mentioned earlier, two effects were produced by the wall coating layer: a slight reduction in the effective flow area and a drastically increased hydrodynamic roughness (*k*_s_). The reduction in the flow area was taken into account through the average thickness of the wall coating layer (*t*_c_), which is a physical parameter that can be measured directly. The hydrodynamic roughness (*k*_s_) value corresponding to each combination of viscous wall coating thickness (*t*_c_), and water Reynolds number was determined using the CFD-based procedure describe above. The results were used to develop a correlation between *k*_s_ and *t*_c_.

### Rectangular flow cell results

**Fig. 6:**
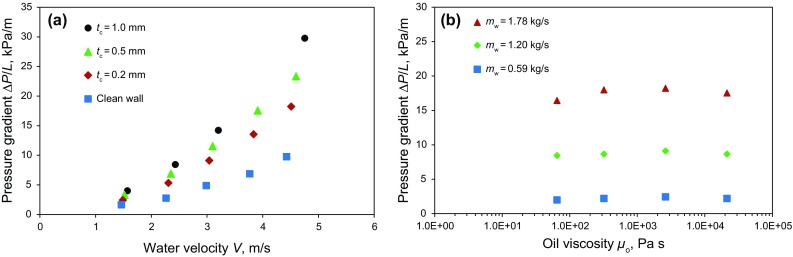
Experimental results for the rectangular flow cell. **a** Pressure gradient (∆*P*/*L*) as a function of bulk water velocity (*V*) and oil coating thickness (*t*_c_) (*µ*_o_ = 2620 Pa s). **b** Effect of water flow rate (*m*_w_) and oil viscosity for a fixed coating thickness (*t*_c_ = 0.2 mm)

The effect of wall coating thickness (*t*_c_) on the measured pressure gradient, for tests involving a specific oil (*µ*_o_ = 2620 Pa s), is demonstrated in Fig. 6a. It can be seen from the figure that operation at higher average velocities (*V* = *m*_w_ */*(*ρ*_w_ *A*_eff_)) causes the pressure gradients (∆*P/L*) to increase approximately with *V* ^2^, as would be expected for the turbulent flow of water through a channel or pipe. Note, however, that compared to the clean wall condition, the measured pressure gradients are significantly higher when the wall is coated with oil (*t*_c_ > 0). Clearly, the primary contributor to the measured pressure loss at any velocity is the presence of the oil coating in the flow cell. Although four different oils with viscosities ranging from 65 to 21300 Pa s were tested (see Table 1), the results for any given oil were almost identical to those presented in Fig. 6a (Rushd 2016). In other words, oil viscosity played a negligible role over the range of viscosities tested here. The observation that viscosity of the coating layer (*µ*_o_) had no appreciable effect on the measured pressure gradients (∆*P/L*) is demonstrated in Fig. 6b.

**Fig. 7:**
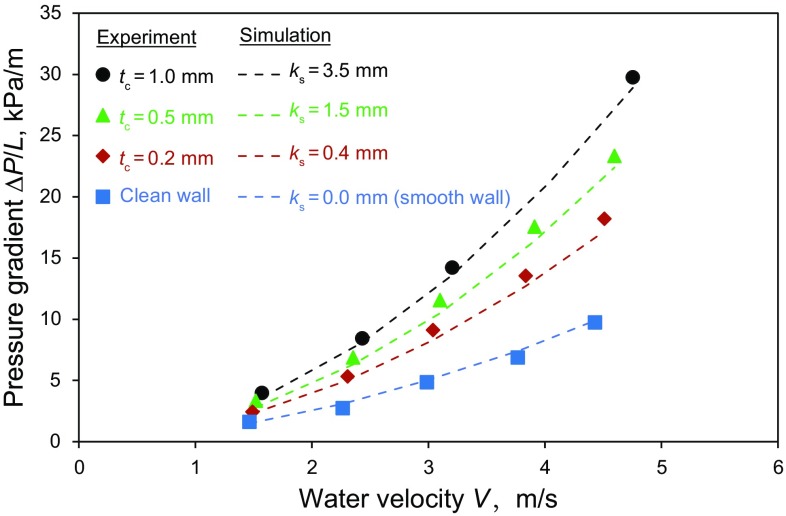
Comparison of simulation and experimental results for the rectangular flow cell (*µ*_o_ = 2620 Pa s)

As can be observed from Fig. 6a, a small increase in coating thickness (*t*_c_) causes a significant increase in pressure gradient (Δ*P*/*L*). The cause of this substantial increase is related primarily to the increase in hydrodynamic roughness of the oil coating layer produced through its interaction with the turbulent water flow through the channel. The coating thickness *t*_c_ reduces *D*_h_ by 0.5%–4% (depending on the value of *t*_c_ tested). If the wall coating layers behaved hydrodynamically as “smooth” surfaces (*k*_s_ = 0), the reduced *D*_h_ would cause a 4%–20% increment in Δ*P*/*L*. The range can be calculated on the basis of Blasius law for a rectangular flow cell (Jones 1976). As Figs. 6a and 7 show, the measured increase in pressure loss with increasing *t*_c_ is in the range of 50%–200%. The substantial increase in Δ*P/L* indicates the importance of the roughness of the coating layer.

In Fig. 7, the measured values are shown in comparison with the predictions obtained from CFD simulations. The simulated pressure gradients agree well with the corresponding measurements when the rectangular flow cell was clean, i.e., when the bottom wall was not coated with oil (*t*_c_ = 0). For these simulations, all four walls of the rectangular flow cell were considered “smooth,” i.e., *k*_s_ = 0. The agreement between the experimental and simulation results indicates the clean walls of the rectangular flow cell to be hydrodynamically smooth. The figure also shows that when the bottom wall was coated with oil, the measured values of Δ*P*/*L* could be accurately predicted. Another important point to note is that the hydrodynamic roughness (*k*_s_) produced by a constant coating thickness (*t*_c_) was not dependent on velocity, for the range of velocities tested here.

### Pipe loop results

**Fig. 8:**
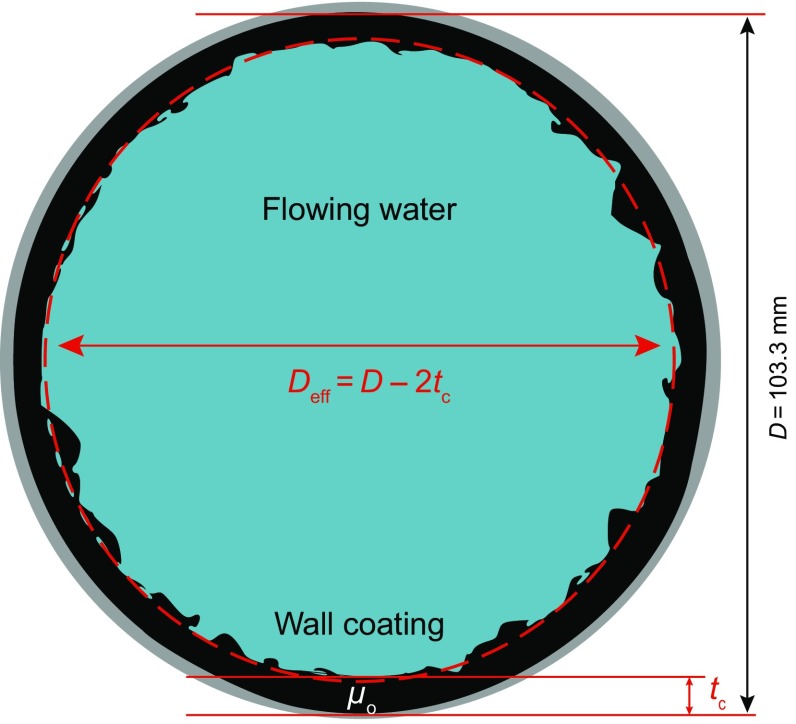
Schematic cross-sectional view of test section in the pipeline

The CFD-based methodology of determining the equivalent hydrodynamic roughness that was developed for the flow cell experiments was then applied to determine the values of *k*_s_ for comparable tests carried out with a recirculating pipe loop. A 103.3-mm (ID) pipe having an internal wall fouled/coated with two different heavy oils (*µ*_o_ ~ 3 and 27 Pa s) was used in experiments. The wall coatings were developed in the course of testing LPF. After completing a set of LPF tests, water at 20 °C was pumped through the pipeline, replacing the oil core. The flow scenario for the pipeline testing is shown schematically in Fig. 8. Pressure loss and wall coating thickness measurements were made simultaneously at mean (bulk) water velocities of *V* = 0.5, 1.0, 1.5, and 2.0 m/s. A custom-built double pipe heat exchanger (Schaan et al. 2002) and a hot film probe were used to obtain wall coating thickness measurements. The wall coating thickness for the pipeline tests decreased with increasing velocity, i.e., *t*_c_ values were dependent on *V* because the coating was partially stripped from the wall as the water velocity was increased. The pressure measurements always reached a steady-state condition at each velocity, which allowed for the calculation a steady-state value of *k*_s_. A more detailed description of the apparatus and test procedure is provided by McKibben et al. (2016).

As was done for the rectangular flow cell tests, CFD simulations of the pipe loop tests were conducted to determine the equivalent hydrodynamic roughness (*k*_s_). A typical comparison of the measured pressure gradients and those obtained from simulations for the pipeline tests are shown in Table 2. Only the results for the higher-viscosity oil (*µ*_o_ ~ 27 Pa s) are shown, as the trend is similar for the other oil (*µ*_o_ ~ 3 Pa s). Because of the way the pipe loop experiments were conducted, different values of *t*_c_ were tested at different water velocities. As the results in Table 2 show, the agreement between the measured pressure gradients and the values determined using the CFD methodology (where *k*_s_ is set by trial and error) is excellent.

**Table 2:** Comparison of equivalent hydrodynamic roughness for pipeline tests (*µ*_o_ ~ 27 Pa s)

Water velocity *V*, m/s	Wall coating thickness *t*_c_, mm	Pressure gradient Δ*P/L*, kPa/m		Hydrodynamic roughness *k*_s_, mm	
		Measured	CFD method	Colebrook correlation	CFD method
1.0	2.0	0.45	0.45	5.9	5.5
1.5	1.4	0.81	0.84	4.1	3.5
2.0	0.8	1.10	1.17	2.5	2.0

The values of *k*_s_ for the pipeline tests were corroborated by estimating the same values on the basis of the Colebrook correlation:

1 $$\frac{1}{\sqrt f } = - 2\log_{10} \left( {\frac{{k_{\text{s}} }}{{3.7D_{{\text{eff}}} }} + \frac{2.51}{{Re_{\text{w}} \sqrt f }}} \right), \quad 4 \times 10^{3} < Re < 1 \times 10^{8}$$

The values of *k*_s_ for these tests were determined using Eq. (1) and the CFD methodology. These are two completely different approaches for determining *k*_s_. The values calculated on the basis of the Colebrook formula agree reasonably well with those obtained with the CFD method. The results from the two calculation methods are presented in Table 2 for the higher-viscosity oil (~27 Pa s). Similar agreement was found when comparing the two calculation methods for the 3 Pa s oil coating as well.

### Correlation development

A correlation between *k*_s_ and *t*_c_ is proposed here, on the basis of the rectangular flow cell data and the pipe flow tests described previously:

2 $$k_{\text{s}} = 2.76t_{\text{c}}, \quad 0.2 \,\,{\text{mm}} \le t_{\text{c}} \le 2.0 \,\,{\text{mm}}$$

**Fig. 9:**
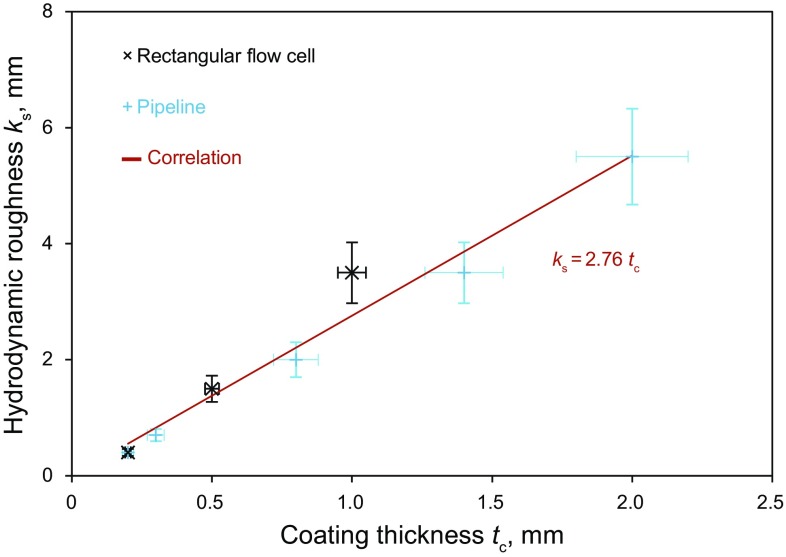
Correlation between hydrodynamic roughness (*k*_s_) and coating thickness (*t*_c_)

The correlation is illustrated in Fig. 9. The proportionality constant of the equation is determined with a regression analysis for which *R* ^2^ = 0.96. The average uncertainty associated with the predictions of this correlation is ±14%. The data set used for developing the correlation set presented in Table 3.

**Table 3:** Data used to develop the correlation between *k*_s_ and *t*_c_ (Eq. 2)

Apparatus	Hydraulic diameter *D*_h_, mm	Oil viscosity *µ*_o_, Pa s	Average velocity *V*, m/s	Coating thickness *t*_c_, mm	Hydrodynamic roughness *k*_s_, mm
Rectangular flow cell	20	65, 320, 2 620, 21 300	1.5	0.2	0.4
			3.1		
			4.5		
		2 620, 21 300	1.5	0.5	1.5
			3.1		
			4.6		
			1.6	1.0	3.5
			3.2		
			4.8		
Pipeline	100	3	1.0	0.2	0.4
			1.5	0.3	0.7
		27	1.0	0.8	2.0
			1.5	1.4	3.5
			2.0	2.0	5.5

As shown in Fig. 9, eight data points were used to develop the correlation. Three of these points were obtained from the experiments conducted with the flow cell, and five points were obtained from pipeline tests. Multiple combinations of oil viscosity, water flow rate, and coating thickness were used for the flow cell experiments. Therefore, the three data points for the rectangular flow cell actually correspond to 24 different flow conditions, meaning the correlation is based on 29 distinct flow conditions.

The relationship between *k*_s_ and *t*_c_ proposed in this work is the first of its kind. To the best of our knowledge, a similar correlation is not available in the literature. An example of its application to predict pressure losses in a fouled/coated pipeline is presented in Appendix 2. Using the correlation in its current form is subject to the knowledge of coating thickness (*t*_c_), which is possible to measure for a LPF system (Schaan et al. 2002). Additional efforts are currently underway to correlate *t*_c_ with flow parameters so that its direct measurement will no longer be necessary.

## Summary

The objective of the present study is to provide detailed information about the hydrodynamic roughness that a wall coating of viscous oil produces. The results reported here can be summarized as follows:

1. A film of viscous wall coating substantially increases the measured pressure loss, primarily as a result of the rough/rippled structure that forms on the surface of the coating, which produces a very large value of the equivalent hydrodynamic roughness, *k*_s_.
2. Experiments were conducted using two different geometries—a rectangular flow cell and a 100-mm (diameter) pipeline loop—and using different oils to produce the wall coating layer. The oil viscosities spanned four orders of magnitude (3 ≤ *μ*_o_ ≤ 2.1 × 10^4^ Pa s). Water alone (i.e., no oil in the bulk flow) was circulated through both test cells under highly turbulent conditions (2.9 × 10^4^ < *Re*_w_ < 2 × 10^5^).
3. The results obtained from the two test geometries were in very close agreement and showed that the hydrodynamic roughness produced by a wall coating of viscous oil was essentially independent of oil viscosity. In fact, the thickness of the coating layer directly determines its hydrodynamic roughness.
4. A new correlation was proposed to relate the hydrodynamic roughness produced by a viscous wall to the thickness of the coating layer. The correlation will be a critical component of any advance model of lubricated pipe flow with wall fouling.

## Appendix 1: description of *ω*-RSM

Most important features of the *ω*-RSM are described here on the basis of Fletcher et al. (2009) and ANSYS CFX-Solver Theory Guide (2010). In this narrative, the differential equations are presented with index notation^1^. The Reynolds-averaged Navier–Stokes (RANS) equations of continuity and momentum transport for an incompressible fluid can be presented with following equations.

Continuity:

3 $$\frac{{{\text{D}}\rho }}{{{\text{D}}t}} = 0$$

Momentum transport:

4 $$\rho \frac{{{\text{D}}U_{i} }}{{{\text{D}}t}} = - \frac{\partial }{{\partial x_{i} }}\left[ {p + \frac{2}{3}\mu \frac{{\partial U_{k} }}{{\partial x_{k} }}} \right] + \frac{\partial }{{\partial x_{j} }}\left[ {\mu \left( {\frac{{\partial U_{i} }}{{\partial x_{j} }} + \frac{{\partial U_{j} }}{{\partial x_{i} }}} \right)} \right] - \rho \frac{\partial }{{\partial x_{j} }}\left( {\tau_{ij} } \right) + S_{i}$$

In Eq. (4), *p* is the static (thermodynamic) pressure; *S*_*i*_ is the sum of body forces, and *τ*_*ij*_ is the fluctuating Reynolds stress contributions.

A number of models are available in ANSYS CFX 13.0 for the Reynolds stresses (*τ*_*ij*_) in the RANS equations. Among the available models, *ω*-RSM is selected as the most suitable for the current work. In this model, *τ*_*ij*_ is made to satisfy a transport equation. A separate transport equation is solved for each of the six Reynolds stress components of *τ*_*ij*_. The differential transport equation for Reynolds stress is as follows:

5 $$\rho \frac{{{\text{D}}\tau_{ij} }}{{{\text{D}}t}} = - \rho P_{ij} - \rho \varPhi_{ij} + \frac{2}{3}\beta '\rho \omega k\delta_{ij} + \frac{\partial }{{\partial x_{k} }}\left[ {\left( {\mu + \frac{{\mu_{t} }}{{\sigma_{k} }}} \right)\frac{{\partial \tau_{ij} }}{{\partial x_{k} }}} \right]$$

The Reynolds stress production tensor *P*_*ij*_ is given by:

6 $$P_{ij} = \tau_{ik} \frac{{\partial U_{j} }}{{\partial x_{k} }} + \tau_{jk} \frac{{\partial U_{i} }}{{\partial x_{k} }}, P = \frac{1}{2}P_{kk}$$

The constitutive relation for the pressure–strain term *Φ*_*ij*_ in Eq. (5) is expressed as follows:

7 $$\varPhi_{ij} = \beta^{\prime}C_{1} \rho \omega \left( {\tau_{ij} + \frac{2}{3}k\delta_{ij} } \right) - \hat{\alpha }\left( {P_{ij} - \frac{2}{3}P\delta_{ij} } \right) - \hat{\beta }\left( {D_{ij} - \frac{2}{3}P\delta_{ij} } \right) - \hat{\gamma }\rho k\left( {S_{ij} - \frac{1}{3}S_{kk} \delta_{ij} } \right)$$

In Eq. (7), the tensor *D*_*ij*_ and the model coefficients are

8 $$D_{ij} = \tau_{ik} \frac{{\partial U_{k} }}{{\partial x_{j} }} + \tau_{jk} \frac{{\partial U_{k} }}{{\partial x_{i} }}$$

Model coefficients:

$$\beta^{\prime} = 0.09$$

$$\hat{\alpha } = \left( {8 + C_{2} } \right)/11$$

$$\hat{\beta } = \left( {8C_{2} - 2} \right)/11$$

$$\hat{\gamma } = \left( {60C_{2} - 4} \right)/55$$

$$C_{1} = 1.8$$

$$C_{2} = 0.52$$

In addition to the stress equations, the *ω*-RSM uses the following equations with corresponding coefficients for the turbulent eddy frequency *ω* and turbulent kinetic energy *k*.

9 $$\rho \frac{{{\text{D}}\omega }}{{{\text{D}}t}} = \alpha \rho \frac{\omega }{k}P_{k} - \beta \rho \omega^{2} + \frac{\partial }{{\partial x_{k} }}\left[ {\left( {\mu + \frac{{\mu_{t} }}{\sigma }} \right)\frac{\partial \omega }{{\partial x_{k} }}} \right]$$

10 $$\rho \frac{{{\text{D}}k}}{{{\text{D}}t}} = P_{k} - \beta^{\prime}\rho k\omega + \frac{\partial }{{\partial x_{j} }}\left[ {\left( {\mu + \frac{{\mu_{t} }}{{\sigma_{k} }}} \right)\frac{\partial k}{{\partial x_{j} }}} \right]$$

11 $$P_{k} = \mu_{t} \left( {\frac{{\partial U_{i} }}{{\partial x_{j} }} + \frac{{\partial U_{j} }}{{\partial x_{i} }}} \right)\frac{{\partial U_{i} }}{{\partial x_{j} }} - \frac{2}{3}\frac{{\partial U_{k} }}{{\partial x_{k} }}\left( {3\mu_{t} \frac{{\partial U_{k} }}{{\partial x_{k} }} + \rho k} \right)$$

Coefficients:

$$\sigma^{*} = 2$$

$$\sigma = 2$$

$$\beta = 0.075$$

$$\alpha = \frac{\beta }{{\beta^{\prime}}} - \frac{{\kappa^{2} }}{{\sigma \left( {\beta^{\prime}} \right)^{0.5} }} = \frac{5}{9}$$

$$\kappa = 0.41$$

$$\sigma_{k} = 2$$

In the previously mentioned transport equations, the turbulent viscosity *µ*_t_ is defined as

12 $$\mu_{\text{t}} = \rho \frac{k}{\omega } .$$

Along with the basic differential equations, the flow near a stationary wall is significant for the turbulent model, *ω*-RSM. Usually a wall is treated with “no-slip” boundary condition for CFD simulations. Mesh-insensitive automatic near-wall treatment is available for the *ω*-RSM implementation in ANSYS CFX 13.0. The treatment is meant to control the smooth transition from the viscous sub-layer to the turbulent layer through the logarithmic zone. Important features of the near-wall treatment for *ω*-RSM are outlined as follows.

(1) In case of a hydrodynamically smooth wall, the viscous sub-layer is connected to the turbulent layer with a log-law region. Velocity profiles for the near-wall regions are as follows:Viscous sub-layer:

13 $$u^{ + } = y^{ + }$$

Log-law region:

14 $$u \, = \, (1/ \, k) \, \ln (y \, ) \, + \, B - \Delta B$$

Here, $u^{ + } = U_{t} /u_{\tau }$, $y^{ + } = \rho \Delta yu_{\tau } /\mu = \Delta yu_{\tau } /v$, and $u_{\tau } = (\tau_{\text{w}} /\rho )^{0.5}$. In the log-law, *B* and Δ*B* are constants. The value of *B* is considered as 5.2 and that of Δ*B* is dependent on the wall roughness. For a smooth wall, Δ*B* = 0. The term Δ*y*, in the definition of *y* ^+^, is calculated as the distance between the first and the second grid points off the wall. Special treatment of *y* ^+^ in CFX allows one to arbitrarily refine the mesh.

(2) For a hydrodynamically rough wall, the roughness is scaled with Nikuradse sand grain equivalent (*k*_s_). Dimensional roughness $k_{\text{s}}^{ + }$ is defined as $k_{s}^{ + } u_{\tau } /v$. A wall is treated hydrodynamically rough when $k_{\text{s}}^{ + }$ is greater than 70. The value of Δ*B* is empirically correlated to $k_{\text{s}}^{ + }$ as follows.

15 $$\Delta B = \frac{1}{\kappa }\ln \left( {1 + 0.3k_{s}^{ + } } \right)$$

Δ*B* represents a parallel shift of logarithmic velocity profile compared to the smooth wall condition.

(3) At the fully rough condition ($k_{\text{s}}^{ + } > 70$), the viscous sub-layer is assumed to be destroyed. Effect of viscosity in the near-wall region is neglected.

(4) The equivalent sand grains are considered to have a blockage effect on the flow. This effect is taken into account by virtually shifting the wall by a distance of 0.5 *k*_s_.

## Appendix 2: sample calculation illustrating application of the proposed correlation

An example illustrating the application of the proposed correlation, i.e., Equation (3) is presented here. The correlation is used to predict the frictional pressure loss for a specific pipe flow case, and then, the predicted value is compared with the measured value. The data for this example are reported by McKibben et al. (2016).

### Measured/known parameters

- Internal diameter of the pipeline (*D*): 103.3 mm
- Average water velocity (*V*): 1.0 m/s
- Density of water (*ρ*_w_): 997 kg/m^3^
- Viscosity of water (*µ*_w_): 0.001 Pa s
- Average thickness of wall coating/fouling (*t*_c_) (measured): 2.0 mm

### Calculations

- Effective diameter (*D*_eff_): $$D_{\text{eff}} = D - 2t_{\text{c}} = 99.3 \,\,{\text{mm}}$$
- Effective velocity (*V*_eff_): $$V_{{\text{eff}}} = V\left( {\frac{D}{{D_{{\text{eff}}} }}} \right)^{2} = 1.1 \,\,{\text{m/s}}$$
- Reynolds number (*Re*_w_): ${Re}_{\text{w}} = \frac{{D_{\text{eff}} V_{{\text{eff}}} \rho_{\text{w}} }}{{\mu_{\text{w}} }} = 1.1 \times 10^{5}$
- Equivalent hydrodynamic roughness (*k*_s_): $k_{\text{s}} = 2.76t_{\text{c}} = 5.52\,\, {\text{mm}}$
- Darcy friction factor (*f*), obtained using the Swamee–Jain correlation: $f = 0.25\left[ {\log \left( {\frac{{k_{\text{s}} }}{{3.7D_{\text{eff}} }} + \frac{5.74}{{Re_{\text{w}}^{0.9} }}} \right)} \right]^{ - 2} = 0.0756$

### Predicted pressure gradient

$$\frac{\Delta P}{L} = f\frac{{\rho_{\text{w}} V_{{\text{eff}}}^{2} }}{{2D_{\text{eff}} }} = 0.44 \,\,{\text{kPa/m}}$$

### Measured pressure gradient

(∆*P*/*L*): 0.45 kPa/m

## List of symbols

*A*_eff_: Effective cross-sectional area (m^2^)
*D*: Internal diameter of the pipeline (m)
*D*_h_: Hydraulic diameter (m)
*D*_eff_: Effective diameter (m)
*f*: Friction factor
*H*: Nominal height of the test cell (m)
*h*_eff_: Effective height (m)
*h*_tp_: Height of the test plate (m)
*k*: Turbulence kinetic energy (m^2^/s^2^)
*k*_s_: Nikuradse sand grain equivalent hydrodynamic roughness (m)
*m*_w_: Mass flow rate of water (kg/s)
*p*: Static (thermodynamic) pressure (Pa)
Δ*P*: Pressure loss (Pa)
Δ*P/L*: Pressure gradient (Pa/m)
*P*_*ij*_: Reynolds stress production tensor
*Re*_w_: Water Reynolds number
*S*_*i*_: Sum of body forces
*T*: Temperature (°C)
*t*_c_: Average thickness of wall fouling/coating layer (mm)
*U*_*i*_: Velocity vector
*V*: Average water velocity (m/s)
*V*_eff_: Effective velocity (m/s)
*δ*_*ij*_: Identity matrix or Kronecker delta function
*τ*_*ij*_: Stress tensor
*ρ*_w_: Water density (kg/m^3^)
*μ*: Viscosity (Pa s)
*μ*_o_: Viscosity of coating oil (Pa s)
*μ*_t_: Turbulent viscosity (Pa s)
*μ*_w_: Water viscosity (Pa s)
*Φ*_*ij*_: Pressure–strain tensor
*ω*: Turbulence eddy frequency
